# Enterovirus 71 vaccine acceptance among parents of children < 5 years old and their knowledge of hand, foot and mouth disease, Chongqing, China, 2017

**DOI:** 10.1371/journal.pone.0225569

**Published:** 2019-11-27

**Authors:** Li Qi, Kun Su, Yu Xia, Wenge Tang, Tao Shen, Qin Li

**Affiliations:** 1 Chongqing Municipal Center for Disease Control and Prevention, Chongqing, China; 2 Chinese Hospital Association, Beijing, China; The University of Hong Kong, CHINA

## Abstract

**Background:**

Enterovirus 71 (EV71) vaccine, which was put into market in China in 2016, has been viewed as a promising prevention measure against severe and fatal hand, foot and mouth disease (HFMD). This study aimed to investigate the knowledge of HFMD and acceptability of EV71 vaccine among parents of under-five in Chongqing, China.

**Methodology /Principal findings:**

A cross-sectional survey was conducted in 2017. A validated questionnaire consisting of three sections including demographic information, knowledge of HFMD, acceptability and reasons for declining vaccination was developed based on literature review. Factors associated with unwillingness to receive EV71 vaccine were explored using multivariate logistic regression. A total of 992 parents finished the questionnaire with a response rate of 91.9%. Awareness of HFMD and EV71 vaccine were reported by 823 (83.0%) parents and 386 (38.9%) parents respectively. Knowledge about HFMD was with a mean score of 5.0 (standard deviation = 3.5) out of a total score of 12. Only 369 (37.2%) participants were classified as with good knowledge level about HFMD. 279 (28.1%) participants had their children received EV71 vaccine and 271 (27.3%) expressed willingness to vaccinate their children after a short-time education about EV71 vaccine. Acceptability of EV71 vaccine increased along with parents’ education level (*p* = 0.008) and HFMD knowledge level (p<0.001). Parents of scattered children had higher acceptability than those of preschool children (*p* = 0.002). 442 (44.6%) of participants were unwilling to have their children vaccinated with EV71 vaccine. The most common reasons for declining EV71 vaccine were doubts about its safety (56.6%) and efficacy (48.3%), and the necessity of vaccination (38.3%). Physicians and vaccination certificate were the parents’ most trusted sources of vaccine information.

**Conclusions:**

Parents’ knowledge about HFMD was not sufficient, and nearly half of the parents expressed unwillingness to vaccinate their children with EV71 vaccine. Our findings stress that more efforts by health authorities in Chongqing are needed to increase the acceptability of EV71 vaccine, especially among parents of preschool children with lower education level.

## Introduction

Hand, foot and mouth disease (HFMD) is an infectious disease caused by a group of enteroviruses [1]. Most HFMD cases are mild and self-limited; however, some cases rapidly develop serious complications such as meningitis and encephalitis, which can be fatal [2]. Infection with enterovirus 71 (EV71) is of particular concern as it is responsible for most of the severe and fatal HFMD cases, particularly in the Asia-Pacific region [3]. In China, EV71 accounted for more than 90% of laboratory-confirmed fatal HFMD cases between 2008 and 2013 [4]. Moreover, EV71 is also associated with many severe diseases in young children (aged < 5 years), including aseptic meningitis and encephalitis [5, 6]. To date, there is no effective antiviral medications to protect against EV71-associated diseases [7, 8]. The preventive measures suggested by World Health Organization are good hygiene, frequent hand washing and social distancing, which seem to have a limited effect on EV71-associated HFMD control and prevention [7, 9]. EV71 vaccine has been viewed as a promising prevention measure against severe and fatal HFMD [10]. Three inactivated EV71 vaccines were licensed by the Chinese Food and Drug Administration (FDA) in December 2015 and recommended for children [11–13]. However, limited data have been collected on the acceptability of EV71 vaccine among parents since the approval of the vaccine by Chinese government.

Chongqing, the largest municipality under direct control of the national government in China, is located in the southwestern China, which is one of the most seriously affected areas by HFMD in China [14]. EV71 vaccine has been put into market in Chongqing since 2016. The aim of this study was to assess parents’ knowledge, attitudes and practices regarding HFMD and EV71 vaccination in Chongqing and to identify potential factors influencing EV71-vaccine uptake.

## Materials and methods

From July 17 to August 14 in 2017, we conducted a cross-sectional survey in 18 primary health centers in Chongqing. A total of 1,080 parents were selected via a three-stage sampling method. At stage 1, nine districts/counties were randomly selected from 39 districts/counties of Chongqing. At stage 2, two primary health centers (PHCs) were randomly selected from each of the sampled district/county, resulting in 18 selected PHCs. At the final stage, 60 parents of children under 5 who attended the PHCs to have their children vaccinated with EV71 vaccine or other vaccines, were selected and invited to participate in a face-to-face interview in each PHC during the proposed study period. The first parent was selected randomly and then the subsequent parents were selected contiguously till the completion of the required sample size. We excluded those parents whose children had a history of suffering HFMD.

The questionnaire was developed by the research team based on literature review [15–16] and tested in a pilot study. The questionnaire included three parts: (1) demographic characteristics, such as age, gender, education level, number of children, and monthly family income; (2) awareness and knowledge of HFMD and EV71 vaccine; (3) acceptability and reasons for not accepting EV71 vaccination.

Awareness of HFMD and EV71 were assessed with the questions “Before today, had you heard of HFMD?” and “Before today, had you heard of EV71 vaccine?”. Parents who answered “yes” to the question were considered to be aware of HFMD and EV71 vaccine. Those who were not aware of EV71 vaccine were given basic information on EV71 vaccine by the interviewer prior to continuing the interview.

Knowledge of HFMD was collectively assessed with 12 single choice question with items of “yes”, “no”, and “don’t know”. Each correct answer was given one point, and each incorrect answer or “don’t know” was given zero point. The total knowledge score ranged from 0 to 12. Finally, all respondents were divided into two groups based on their total knowledge score: good (≥ 8 correct out of 12) and poor (< 8 correct out of 12).The questions “have you already vaccinated your children with EV71 vaccine?” and “are you willing to vaccinate your children with EV71 vaccine?” were used to assess the acceptability of EV71 vaccine. Parents who answered yes to either of the two questions were considered to have good willingness to take EV71 vaccine. For parents who answered “no” to both questions were further asked to give their reasons for not accepting vaccine with a multiple choice question with 5 specified reasons and one open choice (“Others, please specify”).

We conducted a pilot survey with a small sample of participants (n = 50) to test the acceptability and feasibility of the questionnaire. Based on the results of the pilot survey, some minor wording changes were made in the revised version, which was then used in this study.

## Statistical analysis

Descriptive statistics were obtained for all survey items. The Chi-square tests were used for comparisons between categorical variables. Univariate analysis was conducted to compare factors influencing acceptability of EV71 vaccinations, for those factors with statistical significance (*p* < 0.05) were included in multivariate logistic regression analysis. SPSS Statistics software (Release 25.0, SPSS Inc, and IBM Company, Chicago, IL, USA) was used for all of the statistical analyses.

### Ethical approval

The study protocol and the verbal informed consent process were approved by the Ethic Committee of Chongqing Center for Disease Control and Prevention. Verbal consent was obtained from all participants-parents. All data analyzed anonymously.

## Results

### Demographics

A total of 1, 080 parents were invited to participate in the survey. Of them, 992 (91.9%) parents completed the questionnaires and were included in the analysis, while 88 parents refused. Demographic characteristics of participants were summarized in Table 1.

**Table 1:** Demographic characteristics of participants in the study of knowledge of HFMD and acceptability of EV71 vaccine among parents of under-five in Chongqing, China.

Characteristics	Number	Percentage (%)
Total number Relationship	992	
Father	319	32.2
Mother	673	67.8
Level of education		
Primary school or below	167	16.8
Junior high or middle school	239	24.1
High school or above	576	58.1
Monthly family's income		
Low	73	7.4
Median	442	44.5
High	444	44.8
Refuse to answer	33	3.3
Children's gender		
Boy	499	50.3
Girl	493	49.7
Children's category*		
Scattered children	806	81.2
Preschool children	186	18.8
Number of children in the family		
One	714	72.0
≥ Two	278	28.0
Parents’ age		
20~29 years old	522	52.6
30~39 years old	411	44.5
40 years old and above	29	2.9
Children’s age		
Less than 1 years old	408	41.1
1~ years old	235	23.7
2~ years old	163	16.4
3~ years old	100	10.1
4~ 5 years old	86	8.7

*Note: Scattered children are defined as children whose cares were given by their family members and generally younger than 3 years old. Preschool children are classified as those children who are enrolled in the kindergarten or the nursery, who are usually older than 3 and younger than 5 in this study.

Of the 992 participants, 67.8% (673/992) were mothers and 32.2% (319/992) were fathers with the mean age of 28.5 (range: 20–44 years). Most commonly reported education level was high school or above (58.1%, 576/992). Of the 992 children who were vaccinated on the survey days, 50.3% (499/992) were boys and 49.7% (493/992) were girls with mean age of 1.6 (range: 1 month– 5 years old). Majority of them (81.2%, 806/992) were scattered children and 18.8% (186/992) were preschool children.

### Awareness and knowledge of HFMD

Most (83.0%, 823/992) of the participants had heard of HFMD before the investigation. However, their knowledge regarding the characteristic symptoms, infectiousness, prevention measures and transmission routes of HFMD varied significantly (Table 2). Indeed, 75.1% of them knew HFMD is infectious and characterized by non-itchy red rash with blisters on the hands and feet and painful mouth ulcers (60.4%), whereas the awareness of other aspects was poor. Particularly, less than one third of participants knew the transmission routes of HFMD.

**Table 2:** Knowledge of HFMD among parents (n = 992) of children < 5 years old in Chongqing, China.

Items	Number	Percentage (%)
**HFMD is an infectious disease**	745	75.1
**Symptoms of HFMD**	599	60.4
**HFMD is caused by intestinal tract viruses**	437	44.1
**Epidemic seasons**	336	33.9
**Preventive Measures**		
Bask the quilts and clothes	523	52.7
Avoid contacting with HFMD patients	535	53.9
Frequently hand washing	423	42.6
Sterilizing towels, toys, etc.	246	24.8
**Routes of Transmission**		
Contact with the feces of an infected person	316	31.9
Contact with the mucous membrane when an infected individual coughs or sneezes	275	27.7
Contact with the secretion or fluid from blisters of an infected person	271	27.3
Contact with contaminated surfaces, e.g. towels, toys	266	26.8

The mean score of HFMD knowledge was 5.0 (SD = 3.5) out of 12. A total of 369 (37.2%) participants were classified as with good knowledge related to HFMD, 454 (62.8%) were classified as having poor knowledge (Table 3).

**Table 3:** HFMD knowledge score of participants in study of knowledge of HFMD and acceptability of EV71 vaccine among parents of under-five in Chongqing, China.

HFMD knowledge score	Participants (%)
Good HFMD knowledge (≥ 8 correct answer out of 12 questions)	369 (37.2)
Poor HFMD knowledge (< 8 correct answer out of 12 questions)	623 (62.8)

### Information about EV71 vaccine

**Fig 1:**
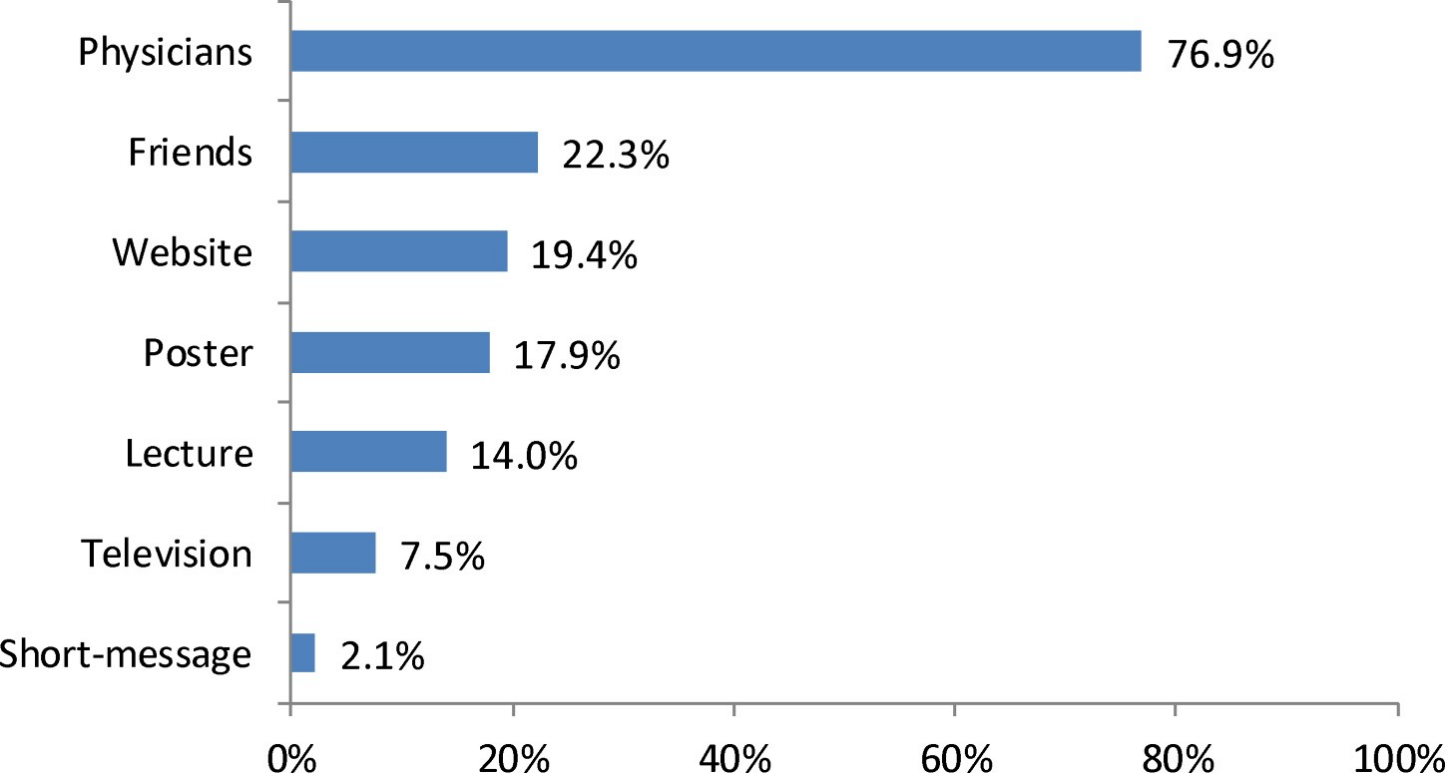
EV71 vaccine information sources of 386 participants. The blue columns indicate the proportions of participants got information from each source.

Only 386 (38.9%) participants had heard of the EV71 vaccine before, and most got the information about EV71 vaccine from their physicians (Fig 1).

**Fig 2:**
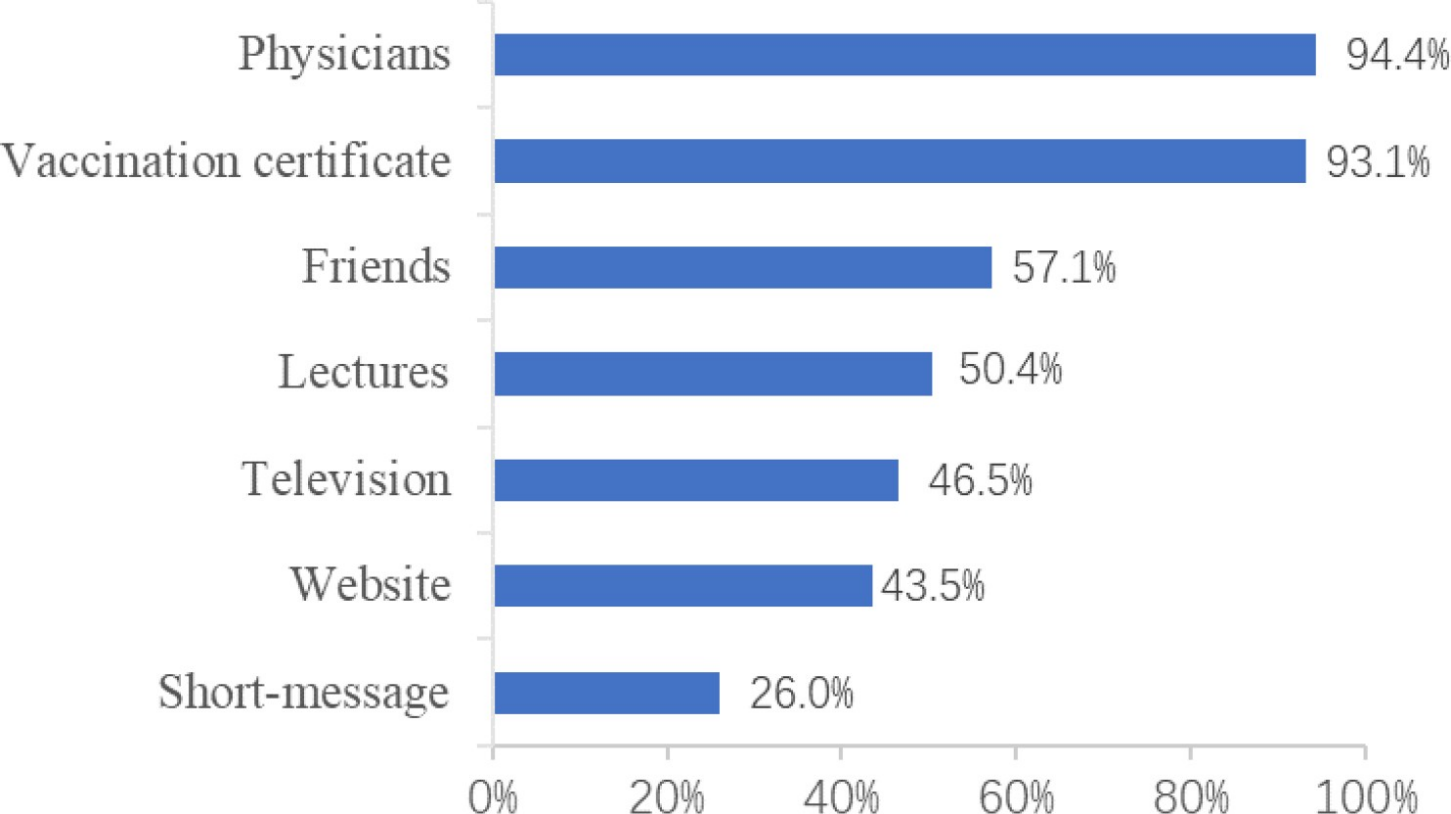
The credibility of different vaccine information sources of participants (n = 992). The blue columns indicate the participants’ credibility of each vaccine information source.

Regarding their most trusted vaccine information source, the majority of participants preferred physicians (94.4%, 936/992) and vaccination certificate (93.1%, 924/992), followed by friends (57.1%, 566/992) and lectures (50.4%, 500/992), while other information channels were considered as less credible (Fig 2).

### Acceptability of EV71 vaccine

**Fig 3:**
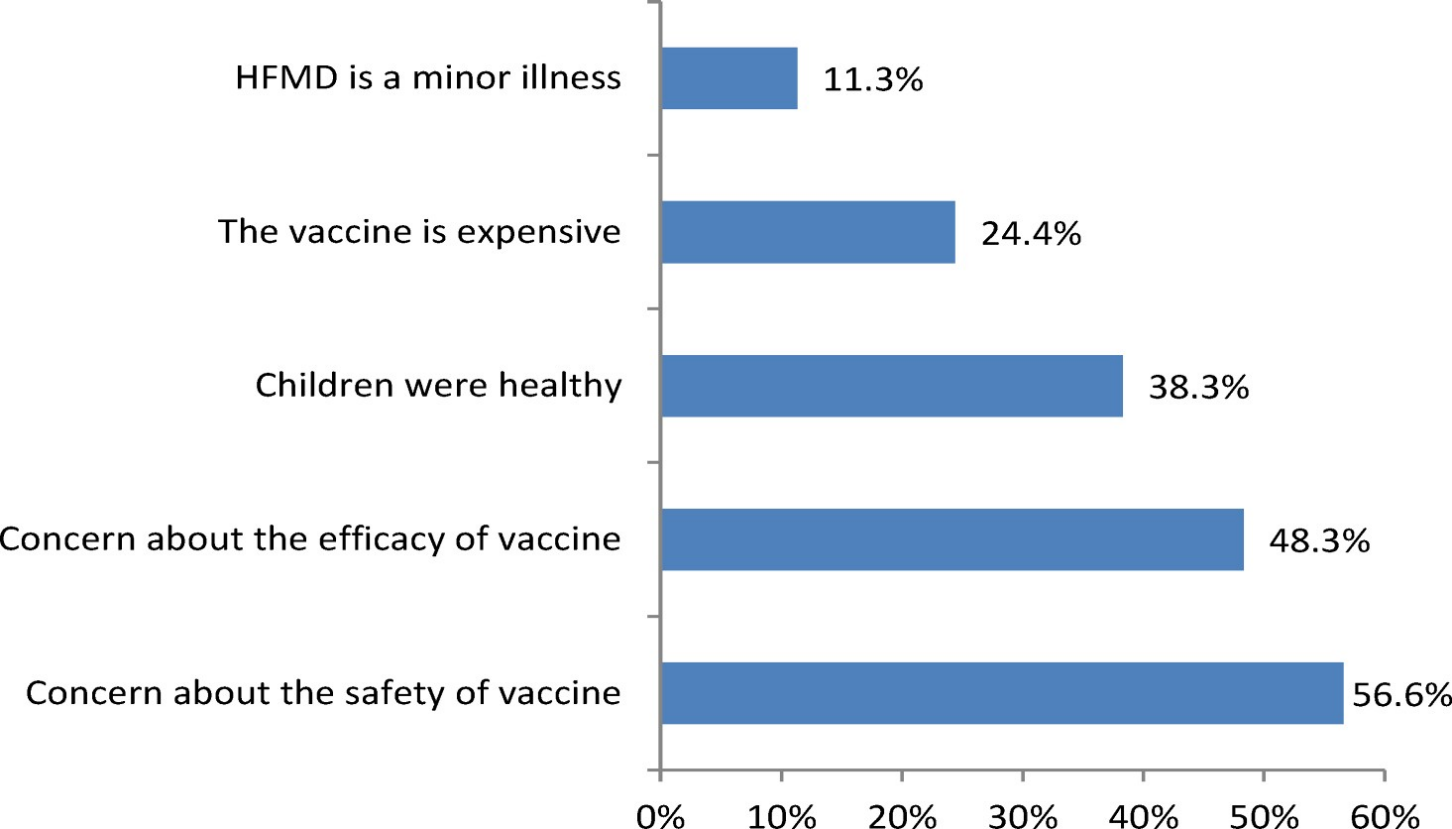
Participants’ reasons for declining EV71 vaccination. The blue columns indicate the proportions of possible reasons for declining EV71 vaccination.

A total of 279 participants (28.1%) had already vaccinated their children with EV71 vaccine by the survey time and 271 (27.3%) expressed willingness to vaccinate their children after a short-time education about EV71 vaccine during the investigation. However, 442 (44.6%) participants declined vaccination. There were a few of possible reasons for unwillingness to vaccinate their children with EV71 vaccine: 56.6% (250/442) respondents concerned about the safety of the vaccine, and 48.3% (213/442) concerned about its efficacy. In addition, 38.3% (169/442) doubted its necessity. 24.4% (108/442) of the participants thought that the vaccine too expensive, and 11.3% (50/442) considered HFMD as a mild illness (Fig 3).

The characteristics of participants who accepted EV71 vaccine and those who declined EV71 vaccine were compared by χ^2^ test and presented in Table 4. In univariate analysis, the following factors were statistical significantly associated with the acceptability: parents’ education level and knowledge of HFMD, monthly family income and children's category. All covariables were included in the multiple logistic regression model.

**Table 4:** The acceptability of EV71 vaccination and uni-variate analysis in study of knowledge of HFMD and acceptability of EV71 vaccine among parents of under-five in Chongqing, China.

Variables	Number	Acceptance (%)	χ^2^	p value
Relationship				
Farther	319	173 (54.2)	0.28	0.597
Mother	673	377 (56.0)		
Parents’ education level				
Primary school or below	167	77 (46.1)	10.48	0.008
Junior high or middle school	239	127 (53.1)		
High school or above	576	346 (60.1)		
Monthly family income				
Low	73	41 (56.2)	8.63	0.035
Median	442	224 (50.7)		
High	444	268 (60.3)		
Refuse to answer	33	17 (51.5)		
Children's gender				
Boy	499	271 (54.3)	0.52	0.429
Girl	493	279 (56.6)		
Children's category				
Scattered children	806	466 (57.8)	9.30	0.002
Preschool children	186	84 (45.2)		
Number of children in the family				
One	714	400 (56.0)	1.83	0.557
≥ Two	278	150 (54.0)		
Knowledge of HFMD				
Poor	386	289 (74.9)	17.24	<0.001
Good	606	261 (43.1)		
Children’s age				
Less than 1 years	408	233 (57.1)	23.15	<0.001
1~ years	235	122 ((51.9)		
2~ years	163	111 (68.1)		
3~ years	100	51 ((51.0)		
4~ 5 years	86	33 (38.4)		
Parents’ age				
20~29 years old	522	295 (56.5)	0.52	0.77
30~39 years old	441	239 (54.2)		
40 years old and above	29	16 (55.2)		

From the multiple logistic regression analysis, children’s category, parents’ education level and knowledge of HFMD were significantly and independently associated with the acceptability of EV71 vaccine after adjusting for the other variables in the model (Table 5). Parents of scattered children were 1.79 times more likely to vaccinate their children (OR = 1.79, 95CI: 1.29–2.50) compared to those of pre-school children. Parents with education level of high school or above were 1.54 times more likely to vaccinate their children (OR = 1.54, 95CI: 1.08–2.21) than those with education level of primary school or below. Parents with good knowledge of HFMD were 1.67 times more likely to vaccinate their children (OR = 1.67, 95CI: 1.27–2.20) compared to those with poor knowledge of HFMD.

**Table 5:** Factors potentially influencing the acceptability of EV71 vaccine among participants in study of knowledge of HFMD and acceptability of EV71 vaccine among parents of under-five in Chongqing, China.

Variables	Acceptability of EV71 vaccine		
	Adjusted OR	95% CI	p-value
**Children's category**			
Preschool children	Ref		
Scattered children	1.79	1.29–2.50	0.001
**Education level**			
Primary school or below	Ref		
Junior high or middle school	1.27	0.85–1.89	0.252
High school or above	1.54	1.08–2.21	0.018
**Knowledge of HFMD**			
Poor	Ref		
Good	1.67	1.27–2.20	<0.001
**Monthly family income**			
Low	Ref		
Median	0.80	0.52–1.24	0.312
High	1.16	0.75–1.81	0.496

## Discussion

EV71 vaccines have been put into market in China since 2016 and have been viewed as a promising prevention measure against severe and fatal HFMD [12, 17]. The objective of this study was to provide useful information for further research on HFMD vaccine and immunization strategy of the EV71 vaccine. The result indicated a suboptimal EV71 vaccine acceptance among parents of under-five in Chongqing, China. Only 28.1% of participants had their children vaccinated and 44.6% declined even after being given the official EV71 vaccine recommendation statement during the investigation. This coverage rate is lower than other extra expanded immunization program (EPI) vaccines classified as class 2, which are not free of charge, such as chickenpox vaccine, which was 44.7% in urban area of Chongqing [18]. Several factors might be proposed to explain the low vaccine acceptability.

Firstly, lack of propaganda about the EV71 vaccine and the fact that the vaccine was available recently. In this study, only 38.9% of participants had heard of EV71 vaccine before the investigation. The result showed 27.3% of participants expressed willing to vaccinate their children after a short-time education about EV71 vaccine during the investigation. This finding suggests health publicity and education on EV71 vaccine should be enhanced. Regarding the different vaccine information sources, physician was the most credible source, followed by vaccination certificate. Therefore, it would be helpful for doctors to recommend EV71 vaccine to parents and provide them with information about the risks of EV71 infection and benefits of vaccination [19]. It is also advisable to conduct studies to explore the factors associated with recommendation of EV71 vaccination by physicians. Moreover, promotion of EV71 vaccine through vaccination certificate might be also a cost-effective immunization implementation strategy.

Secondly, poor knowledge of HFMD among parents also contributed to the poor acceptance of EV71 vaccine. The mean score of HFMD knowledge was only 5.0 out of 12. The multivariable analysis showed that parents’ knowledge on HFMD was significantly positively associated with acceptability, as was reported by other similar studies [20–22]. Furthermore, parents of higher education level and of scatter children were more likely to vaccinate their children, which is consistent with other studies [23, 24]. The findings suggest that enhancing public education on HFMD knowledge might improve their willingness to vaccinate their children, and the information needs to be communicated in simplified language for parents with lower education level. EV71 vaccination among children is one of the most effective means of preventing HFMD transmission in such places as kindergartens and nurseries, where the virus can be rapidly spread. Therefore, it is advisable to enhance health publicity and education on HFMD and EV71 vaccine in these places.

Thirdly, lack of confidence in the safety and efficacy of the EV71 vaccine directly lead to a low acceptance, which is similar to previous studies on other vaccines [25, 26]. The top two reasons against vaccination were “concern about the safety of vaccine” and “concern about the efficacy of vaccine”, accounting for 56.6% and 48.3% of those participants unwilling to vaccinate their children. It reflected the misconception about EV71 vaccine among parents. Nowadays, in China, there is an irrational panic and wide-spread concern about vaccination as result of many misreported adverse events about vaccination [27]. When a new vaccine such as EV71 vaccine is licensed for use, the people become more cautious about it. Therefore, future health education campaigns and messages should be aimed at easing concerns about safety and effectiveness of EV71 vaccine in parents.

There is no single intervention strategy that works for all aspects of vaccination hesitancy. The tailed strategies to improve vaccine acceptability should be developed based on the different areas and vaccines. According to the results in our study, we can improve the acceptability of EV71 vaccine from the following three aspects. Firstly, HFMD knowledge level and awareness of EV71 vaccine of the target parents must be improved. Appropriate incentives should be given to physicians to increase their involvement [28]. The contents of the health education should include those aspects which parents with poor knowledge, such as the routes of transmission. Secondly, people’s concerns about safety and efficacy of EV71 vaccine should be echoed. To help address misconceptions among parents, tailed educational messages should be targeted at parents. Thirdly, strong physician recommendation and promotion through vaccination certificate were key facilitators to optimize uptake of EV71 vaccine in this region.

Our study could be interpreted with some limitations. Firstly, the convenient samples of parents who attended the PHCs to vaccinate their children during the proposed study period may not adequately represent the population in Chongqing. Secondly, the cross-sectional nature of this study limits the extent to which we can evaluate changes in KAP across time. Finally, the EV71 vaccination status of children was self-reported and not verified through records.

## Conclusions

In conclusion, parents’ knowledge about HFMD was not sufficient, and nearly half of parents expressed unwillingness to vaccinate their children with EV71 vaccine in Chongqing. Misconceptions about EV71 vaccination were prevalent among parents. Our findings stress that more efforts by health authorities in Chongqing are needed to increase the acceptability of EV71 vaccine, especially for parents of preschool children with lower education level.

## Supporting information

S1 Database Database of the study. (SAV)

## Abbreviations

HFMD: Hand, foot and mouth disease
EV71: Enterovirus 71
FDA: Food and Drug Administration
PHC: Primary health centers
SD: Standard deviation
